# Effect of Temperature Gradient and Cooling Rate on the Solidification of Iron: A Molecular Dynamics Study

**DOI:** 10.3390/ma17246051

**Published:** 2024-12-11

**Authors:** Qin Qin, Weizhuang Li, Wenrui Wang, Dongyue Li, Lu Xie

**Affiliations:** School of Mechanical Engineering, University of Science and Technology Beijing, Beijing 100083, China; qinqin@me.ustb.edu.cn (Q.Q.); lwz199506@163.com (W.L.); gmbitwrw@ustb.edu.cn (W.W.)

**Keywords:** temperature gradient, stress distribution, phase transformation, cooling rate

## Abstract

In this study, molecular dynamics (MD) simulations were employed to compare the effects of different solidification conditions on the solidification behaviour, stress distribution, and degree of crystallization of iron. The results indicate significant differences in nucleation and microstructural evolution between the two solidification methods. In the homogeneous temperature field, the solidification of iron is characterized by instantaneous nucleation. The BCC phase surged at 1431 K followed by the phenomenon of latent heat of crystallization. As the temperature continued to decrease, the percentage of the BCC phase continued to increase steadily. Eventually, the atoms aggregated to form a crystal nucleus and grow outward to form polycrystalline structures. During gradient solidification, continuous nucleation of iron leads to a slow increase in the BCC phase. From the initial stage of solidification, the solid–liquid interface moves in the direction of higher temperature and is accompanied by a higher stress distribution. Furthermore, increasing the temperature gradient, particularly the cooling rate, accelerates the transformation efficiency of iron in the gradient solidification process. In addition, increasing the cooling rate or temperature gradient reduces the residual stress and crystallinity of the solidified microstructure. It is worth noting that an increased temperature gradient or cooling rate will produce higher residual stress and uneven microstructure in the boundary region. This study provides an atomic-level understanding of the improvement in the solidification performance of iron.

## 1. Introduction

The solidification process is affected by various factors such as temperature gradient [1]. These factors lead to differences in the solidification behaviour and microstructure of metals during solidification, such as phase transformation, solute redistribution, etc. These phenomena will further affect the macroscopic properties of metals. Many studies based on experimental observations often use classical nucleation theory (CNT) to analyze the homogeneous nucleation process of metals [2,3,4]. However, complex kinetic and thermodynamic factors make it extremely difficult to predict metals’ solidification behaviour directly. In recent years, computer simulation techniques have gradually been emphasized in metal solidification. The nucleation and growth during the solidification of high-temperature liquid metals can be predicted on different scales by simulation methods such as molecular dynamics (MD), phase field, and cellular automata (CA) [5,6,7].

Among these methods, the MD method can explore the evolution of microstructure, thermodynamics, and equilibrium transport at the atomic scale to achieve accurate regulation of material properties [8]. A large number of scholars have analyzed the solidification behaviour of different metals using MD methods. These studies have dealt with the nucleation growth process of different metals. Chen et al. [9] investigated the solidification behaviour of pure Al at different isothermal cooling rates using the MD method. It was shown that the increase in cooling rate decreases the nucleation rate and reduces the grain size, thus increasing the growth rate of the grains. Pan et al. [10] studied the spontaneous solidification behaviour of gallium (Ga) under different cooling rates and finally prepared Ga with a purity of 99.99999% by artificial intervention. Zhang et al. [11] presented physical images of liquid copper’s microstructure and energy changes at different temperatures based on the MD method. Other scholars have studied the effects of pressure [12], alloy concentration [13], and nanoparticles [14] during solidification on the solidification behaviour. All these findings are significant for understanding and controlling the solidification process. Thus, it can be seen that most studies on solidification are mainly based on the nucleation and growth of the grain in a uniform temperature field. However, the temperature gradient phenomenon that exists in actual processing has been neglected.

A solid–liquid coexistence region exists during the solidification of metals [15,16]. The solid–liquid interface in this region is characterized by the continuous transformation of liquid phases into solid phases [17,18]. In the process, the solid–liquid interface undergoes complex problems such as phase transition, latent heat of crystallization, and solute partitioning due to mechanical and thermal loads [19]. Many scholars have carried out in-depth studies on this problem. Gan et al. [20] established a solid–liquid interface model of Li-Pb using the EAM potential function. They further investigated the nucleation growth process of the crystal microstructure during the solidification process, as well as the influence of the diffusion of Li and Pb atoms on the solid–liquid interface. Nath et al. [21] studied the relationship between the solid–liquid interface moving speed, temperature, and Cu percentage. Gan X et al. [22] investigated the effects of different temperatures on the density and stress at the solid–liquid interface of Fe-Li alloys. It was found that around the solid–liquid interface, very violent oscillatory stress fluctuations occurred. Other scholars have also researched the stress–strain variation laws at the solid–liquid interface during metal solidification by the MD method [23,24,25]. Liu et al. [26] investigated the change rule of the solid–liquid law at the solid–liquid interface of metal iron by utilizing the EAM potential function. However, the mechanism of microstructural evolution in iron under varying solidification conditions remains unclear. Therefore, it is necessary to compare homogeneous solidification and gradient solidification using MD simulations and further investigate the effects of different temperature gradients and cooling rates on iron solidification. This study aims to provide a theoretical basis for optimizing metal solidification process parameters and accurately controlling the solidification behaviour of iron.

In this study, the MD method was employed to compare iron’s solidification behaviour and stress distribution during homogeneous and temperature gradient solidification. Firstly, the differences between the microstructure evolution, phase transition, and stress distribution of iron during homogeneous and temperature gradient solidification were analyzed. In addition, the effects of different temperature gradients and cooling rates on solidification behaviour and stress distribution were studied in detail.

## 2. Methodology

**Figure 1:**
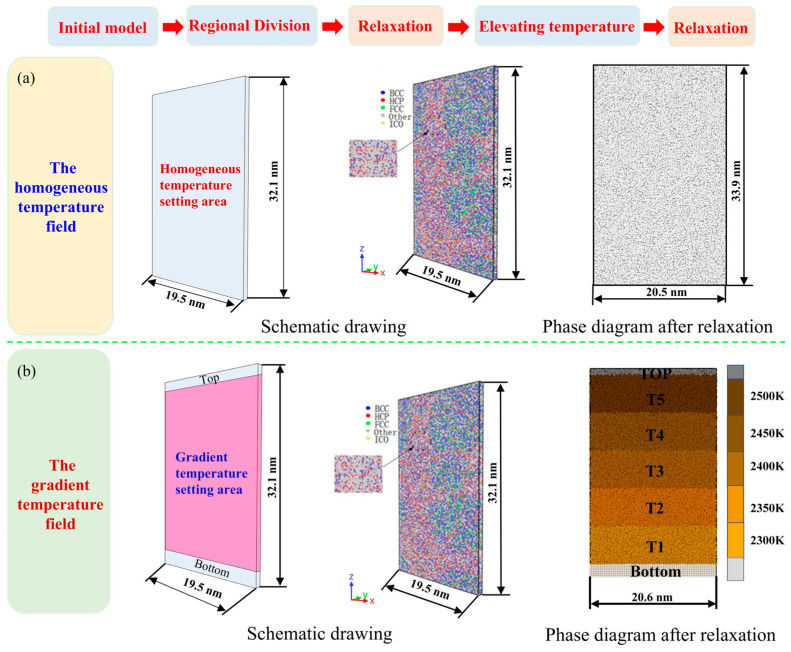
Simulation modelling of (**a**) the homogeneous solidification and (**b**) the gradient solidification of iron.

Figure 1 is a simulation model of two different solidification modes of iron. The volume solidification models have a uniform temperature field. In contrast, the directional solidification models have temperature gradients, and the temperature difference between different regions is equal. Firstly, the initial model with dimensions of 19.5 nm (X) × 0.8 nm (Y) × 32.1 nm (Z) was built. There were 45,696 Fe atoms in the model. In this study, the embedded atom method (EAM) interatomic potential proposed by Mendeleev et al. [27,28] was employed to describe the interactions between Fe atoms. In the simulation, the velocity verlet algorithm [29] was utilized with a timestep of 1 × 10^−15^ s to solve the Newtonian equations of atomic motion. This study utilized the Nose/Hoover thermostat [30] and the Nose/Hoover pressure barostat [31] to control the temperature and pressure. The periodic boundary conditions were adopted in the X, Y and Z directions. All the MD simulations in this study were performed using the large-scale atomic/molecular massively parallel simulator (LAMMPS, v2019) software package [32]. The verification of the model (Figure S1) and the video of the phase transformation (Video S1) are detailed in the Supplementary Materials.

For homogeneous solidification models, the atoms in the initial model had to be equilibrated in the isothermal–isobaric (NPT) ensemble with 0 bar (pressure) and 300 K (temperature), thus allowing the atoms in the model to reach a thermodynamic equilibrium state. Then, the overall model was warmed up to 2500 K (the melting point of pure Fe is 1700 K [33,34,35]). Further, the modelled atoms were relaxed again in the NPT ensemble at 2500 K for 500 ps. Finally, the atoms in the homogeneous solidification model started to solidify at the cooling rate of 4 × 10^9^ K/s.

For the gradient solidification models, it is necessary to divide the gradient temperature region in advance based on the initial model. Therefore, the model was divided into top, bottom, and gradient temperature setting areas along the height direction. The top and bottom regions, which were collectively referred to as the boundary regions, were used to hold the atoms in place. The thickness of the top area (along the *Z*-axis) was 1 nm, and that of the bottom was 2 nm. The gradient temperature setting area was partitioned equally along the *Z*-axis into five regions with a thickness of 6.18 nm. As discussed in Section 3.1, the temperature difference between the regions was 50 K. That is, the temperatures of each region from the bottom to the top were 2300 K, 2350 K, 2400 K, 2450 K, and 2500 K. Similarly, the developed model needed to be relaxed in the NPT ensemble at 0 bar (pressure) and 300 K (temperature). Subsequently, the gradient temperature setting region of the relaxed model was heated up to the target temperature. Further, the gradient solidification model after warming was subjected to the second relaxation process at the corresponding temperature. During the gradient solidification process, the model is solidified at the cooling rate of 4 × 10^9^ K/s, and after 10 cycles, the iron completes solidification. In Section 3.2, the effects of the temperature gradient and cooling rate on the solidification behaviour of iron were systematically investigated. Specifically, the influence of the temperature gradient was analyzed under temperature differences of 50 K, 100 K, 150 K, and 200 K. Similarly, the impact of the cooling rate was examined at cooling rates of 4 × 10^9^ K/s, 1 × 10^10^ K/s, 2 × 10^10^ K/s, and 4 × 10^10^ K/s, respectively.

The Open Visualization Tool (OVITO) [36] was used to visualize and post-process the atomic data from the MD simulations. During the cooling process, common neighbour analysis (CNA) [37] and radius distribution functions (RDFs) [38] were used to assess the phase structure changes and the degree of amorphousness, respectively.

## 3. Results

### 3.1. Solidification at Different Temperature Fields

#### 3.1.1. Solidification Behaviour

**Figure 2:**
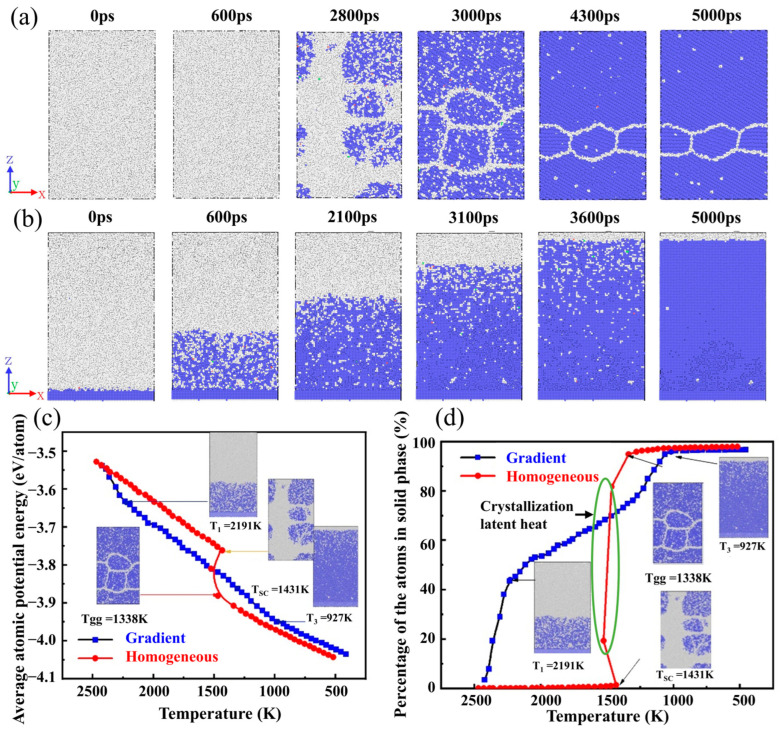
The microstructural transformation of iron during (**a**) homogeneous solidification and (**b**) gradient solidification (blue atoms represent BCC phase; grey atoms represent amorphous phases); (**c**) variation in the average atomic potential energy and (**d**) percentage of atoms in the solid phase with temperature for two solidification conditions.

Temperature plays a critical role in determining the rate and mode of nucleation. Figure 2a presents the microstructure of iron during homogeneous solidification. Within the time range of 0 to 600 ps, no significant solidification is observed in the solidification region. The randomly distributed BCC phase does not begin to form in the region until after 2800 ps. Then, some BCC phases converge to integrate the crystal nucleus, eventually forming a polycrystalline structure with grain boundaries. Figure 2b shows the microstructure of iron during the gradient solidification. From the figure, it is observed that at 0~600 ps, a portion of the amorphous phase transforms into BCC phases. Up to 3600 ps, the percentage of the BCC phase increases gradually. In addition, it is also observed that the solid–liquid interface shape is essentially planar. Up to 5000 ps, most of the amorphous phase transforms into the BCC phase.

Figure 2c shows the average atomic potential energy trend during homogeneous and gradient solidification. During homogeneous solidification, the average atomic potential energy curves change abruptly at 1431 K (2800 ps) and 1338 K (3000 ps). At 1431 K, the system’s average atomic potential energy value decreases rapidly. Combined with the microstructure images, it is believed that the phenomenon is mainly due to the fact that the atoms of iron in the liquid phase begin to transform to the solid phase at this point. It also means that the crystallization nucleation process starts from this temperature [39]. At 1338 K, the decreasing trend of average atomic potential energy within the system slows. This is because when the temperature reaches this point, the nucleation within the system has basically ended [40]. The subsequent process is mainly the growth of grain. During the subsequent solidification process, the motion of the atoms gradually stabilizes and the average atomic potential energy curve steadily decreases. However, the average atomic potential energy change curve of iron during gradient solidification fluctuates less, showing a steady decrease. In addition, atoms in the homogeneous solidification model at the same temperature consistently maintain lower atomic energies in the later stages of solidification. This further indicates that the nucleation rate of iron during gradient solidification is slower than that in homogeneous solidification.

Further, we have statistically varied the curves of the percentage of the atoms in the solid phase during homogeneous solidification, as shown in Figure 2d. During homogeneous solidification of iron, only trace amounts of atoms are converted to the solid phase between 2500 K and 1431 K. But the curve undergoes a sharp and abrupt change at 1431 K. The percentage of the atoms in the solid phase rises abruptly at this time. Combined with the analysis of the microstructure and atomic potential energy changes at this temperature, it is concluded that subcritical nuclei begin to form in iron at this time. As the curing proceeds further, a amount number of the liquid phase transforms into the solid phase. However, the temperature of the whole model shows an increasing and then decreasing trend. This phenomenon is because the crystal nucleus formed at this time are still unstable. Although most of the atoms in the solid phase in the system aggregate to form the crystal nucleus, some of the atoms redissolve to become liquid-phase. That is, the latent heat of crystallization causes the system temperature to rise back up [41]. When the model temperature falls back to 1431 K again, the amount of atoms in the solid phase increases further. At this time, the crystal nucleus has been moulded and the nucleation mode of solidification is transformed into supercritical nucleation [42]. Multiple crystal nucleus begin to appear and grow in the model. When the temperature drops to 1338 K, the nucleation of iron is basically over. The microstructure of iron begins to enter the grain growth stage. At this stage, the percentage of atoms in the solid phase grows slowly until the solidification is complete.

In the gradient temperature field, iron solidifies at around 2191 K. When the temperature decreases to 1272 K, the slope of the blue curve in Figure 2d increases from 1.4 fs^−1^ to 2.5 fs^−1^. Eventually, as the temperature decreases to 927 K, the content of the atoms in the solid phase no longer rises and remains at about 96.5%. Therefore, the nucleation mode of iron during gradient solidification is continuous. This solidification behaviour provides conditions for forming single crystals, thus avoiding defects caused by grain boundaries.

#### 3.1.2. Phase Transformation

**Figure 3:**
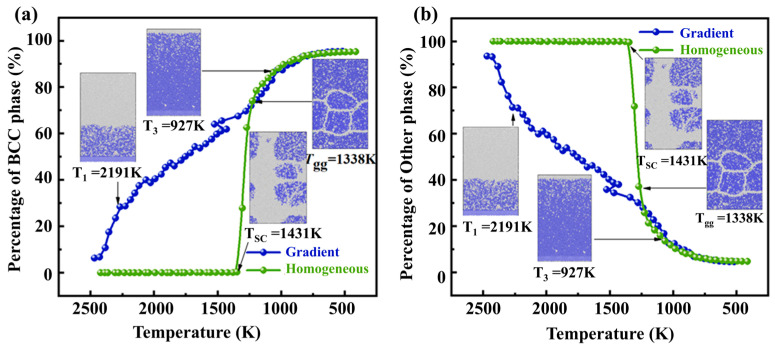
Percentage of (**a**) BCC phase and (**b**) other phases during homogeneous and gradient solidification.

The FCC, BCC, HCP, and other phase structures during the solidification process could be identified by CNA. Generally, the microstructure is identified as liquid or amorphous when a significant proportion of other phases are present in the system. In contrast, the microstructure is identified as solid when a substantial amount of the BCC phase dominates the system [43]. The green curve in Figure 3 shows the percentage of each phase during homogeneous solidification. It can be seen that when the temperature is higher than 1431 K, the model mainly shows the amorphous phase. After the temperature falls below 1431 K, the percentage of the BCC phase rises abruptly. In contrast, the percentage of other phases plummets in the corresponding figure (Figure 3b). This indicates that the transformation of other phase into BCC phase mainly occurs during this process. When the temperature drops to about 1338 K, the percentage of BCC phase increases to 74.07%. The nucleation process is essentially over at this temperature, and the rate of conversion of the BCC phase will show a significant decrease. The growth rate of the BCC phase percentage will slow down in the subsequent solidification process.

The blue curve in Figure 3 illustrates the percentage of each phase during gradient solidification. It can be seen that the percentage of the BCC phase fluctuates and increases while the proportion of other phases decreases. At 927 K, the BCC phase fraction is 87.381%. This means that the transformation of the phase is basically complete. The results show that the amount of this phase generated is essentially the same for both solidification methods. The difference is that in the gradient solidification process, the proportion of the BCC phase is growing all the time, whereas the proportions of the phase compositions can change abruptly at some point during homogeneous solidification.

#### 3.1.3. Effects of Different Temperature Fields on Stress

**Figure 4:**
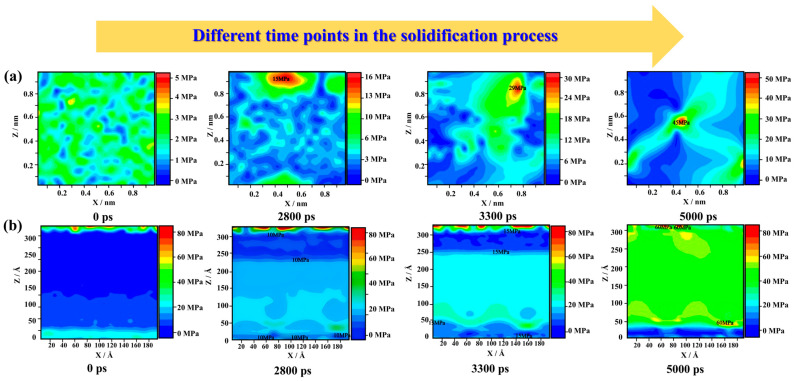
The von Mises stress field of the solidification structures (unit MPa): (**a**) homogeneous solidification and (**b**) gradient solidification.

The von Mises stress field distribution in this paper was determined by the calculation method proposed by Heino et al. [44]. As shown in Figure 4a, the stress values during homogeneous solidification are all below 50 MPa. In the homogeneous temperature field, the initial stress distribution is disordered and the stress values are less than 5 MPa. At 2800 ps, stress concentrations begin to appear in the system as the crystals enter the nucleation and growth state. The maximum stress is increased to 15 MPa. As solidification continues, the stress concentration becomes more pronounced, and the maximum stress further increases to 29 MPa. At 5000 ps, with the previous analysis, it can be shown that the homogeneous solidification produces three grain boundaries. The grain boundary intersection occurs in the region of 50~150 Å and the maximum stress is around 45 MPa. And the stress values inside all three grains are around 10 MPa. As a result, the stress level during homogeneous solidification is low and the stress concentration occurs mainly in the grain boundary intersection region.

The stress distribution during gradient solidification is shown in Figure 4b. At 0 ps, the stresses are all below 5 MPa because the model is filled with atoms of iron in the liquid state. However, the stresses in the boundary regions of the model (top and bottom of the picture) are higher. Therefore, the boundary region and the gradient solidification region should be analyzed differently. From 2800 ps, a clear solid–liquid interface can be observed, and the stresses at the interface are about 10 MPa. Due to the gradual transformation of numerous amorphous phases into the BCC phase from the bottom, it can be seen that the solid–liquid interface always moves in the direction of the negative temperature gradient. The stress values in the 0~250 Å range (along the *Z*-axis) are between 20 MPa and 40 MPa. The stress in the unsolidified part is around 5 MPa. The bottom region has a low stress concentration, which has stresses around 40 MPa. At 3300 ps, the solidification process is basically over, and a dense single-crystal structure is finally formed. At 3300~5000 ps, the overall stress distribution of the model is more uniform, around 40 MPa. There are slight stress concentrations of about 60 MPa in the top and bottom regions. It can be seen that gradient solidification has a more stable overall stress distribution compared to homogeneous solidification. However, the highest stress value in the gradient solidification regions is four times higher than the homogeneous temperature field. The highest stress value in the boundary regions is 1.7 times higher than the stress value in homogeneous solidification.

### 3.2. Influence of Process Parameters

#### 3.2.1. Microstructure Evolution

**Figure 5:**
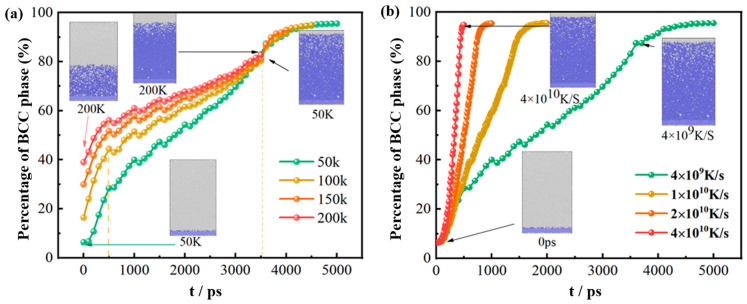
(**a**) The percentage of BCC phase for different temperature differences; (**b**) the percentage of BCC phase for different cooling rates.

In this section, four models with different temperature gradients are developed. The temperature differences between the gradient temperature zones of the four models are set to 50 K, 100 K, 150 K, and 200 K, respectively. The cooling rate is set to 4 × 10^9^ K/s. As shown in Figure 5a, the system with a temperature difference of 50 K has the lowest percentage of BCC phase at 0 ps, which is 6.3%. When the temperature difference between regions increases to 200 K, the percentage of the BCC phase is 39%. This phenomenon indicates that the higher the temperature gradient, the higher the percentage of BCC phase at the beginning of solidification. This phenomenon arises from the correlation where an increased temperature difference results in expanded crystallization regions, thereby augmenting the proportion of the BCC phase [45]. It is further found that the temperature gradient is positively proportional to the increasing rate of BCC percentage before 600 ps. In other words, the larger the temperature gradient, the faster the BCC phase increases. After 600 ps, the increasing rate of the BCC phase percentage starts to decrease at all temperature gradients. At 3600~5000 ps, there is only a slight increase in the percentage of BCC phase at each temperature gradient, and the elevation process is very smooth.

Furthermore, the effect of different cooling rates on the atomic content of the BCC phase was investigated. The selected cooling rates were 4 × 10^9^ K/s, 1 × 10^10^ K/s, 2 × 10^10^ K/s, and 4 × 10^10^ K/s, respectively. The temperature difference is then determined to be constant at 50 K. As shown in Figure 5b, the trends of the percentage curves at different cooling rates are similar and the final BCC phase percentage is basically the same. However, the specific changes during solidification are different. In the initial stage, the percentage of BCC phase increases rapidly due to the presence of the temperature difference. When the cooling rate decreases from 4 × 10^10^ K/s to 4 × 10^9^ K/s, the efficiency of conversion to the BCC phase decreases significantly. Therefore, the cooling rate significantly changes the conversion efficiency but does not affect the final content of BCC structure atoms.

#### 3.2.2. Effect on Stress

**Figure 6:**
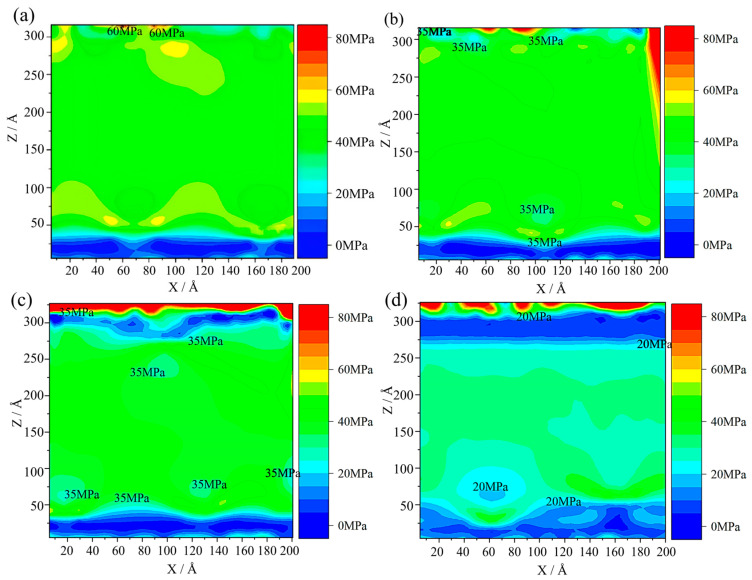
The stress field for different temperature differences (unit MPa): (**a**) 50 K, (**b**) 100 K, (**c**) 150 K, and (**d**) 200 K.

The residual stress in the system after solidification (5000 ps) at various temperature gradients is illustrated in Figure 6. According to Figure 6a, the stress is 40 MPa at the temperature difference of 50 K. The stress concentration in the boundary region is 60 MPa. When the temperature difference is 100 K, the stress in the gradient solidification region decreases to about 35 MPa, representing a reduction of 5 MPa compared to the case at 50 K (Figure 6b). The maximum stress in the boundary region increases to 80 MPa. When the temperature difference increases to 150 K, the value of the stress in the gradient solidification region further decreases to about 30 MPa, while the stress concentration in the boundary region remains at 80 MPa. Moreover, a stratified region with stress levels ranging from 0 to 5 MPa is observed in the middle and upper regions of the model. In Figure 6d, when the temperature difference reaches 200 K, the stress in the gradient solidification region can be seen to be about 20 MPa. Compared to a temperature difference of 50 K, the stress in the gradient solidification region is reduced by 20 MPa, while the stress concentration in the boundary region remains around 80 MPa. It is noteworthy that a stratified region with a stress range of 0 to 5 MPa was observed within the 250~300 Å range (*Z*-axis) of the gradient solidification zone. This region expands significantly as the cooling rate increases. This finding indicates that the higher temperature difference can effectively reduce stress within the solidification region. However, this will also lead to large residual stress and uneven microstructure in the boundary region.

**Figure 7:**
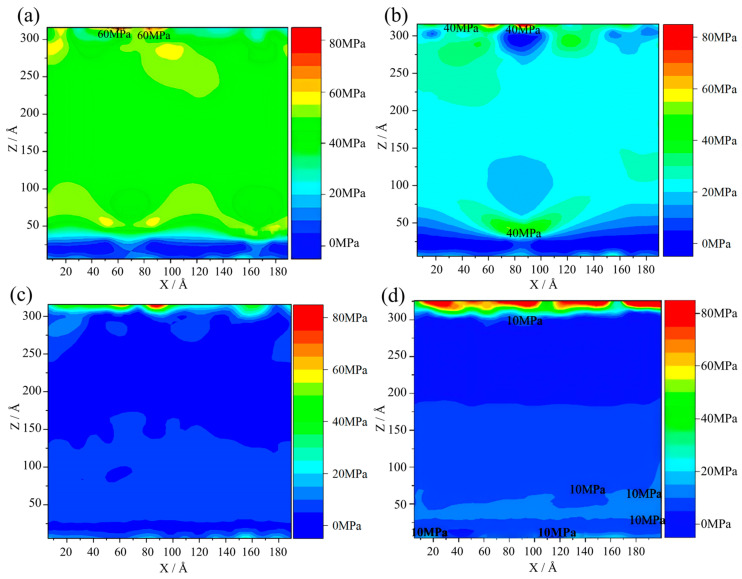
The stress field in solidified structures as a function of the cooling rate (unit MPa): (**a**) 4 × 10^9^ K/s; (**b**) 1 × 10^10^ K/s; (**c**) 2 × 10^10^ K/s; and (**d**) 4 × 10^10^ K/s.

Furthermore, the effect of cooling rate on the residual stress during solidification was investigated, as shown in Figure 7. At the cooling rate of 4 × 10^9^ K/s, the stress in the gradient solidification region is approximately 40 MPa and the stress concentration in the boundary region is about 60 MPa. When the cooling rate increases to 1 × 10^10^ K/s, the stress distribution in the gradient solidification region becomes uniform, decreasing to 20 MPa. In addition, a stress concentration phenomenon occurs in the region of about 80~100 Å (*X*-axis), and the stress value is about 30 MPa. When the cooling rate is 2 × 10^10^ K/s, the overall stress in the solidified microstructure drops significantly to about 5 MPa. The stress concentration is only in the boundary region and is about 20 MPa. At the cooling rate of 4 × 10^10^ K/s, the overall stress remains uniformly distributed at approximately 5 MPa. The stress concentration in the boundary region is about 80 MPa. Additionally, stress layering is observed in the gradient solidification model at cooling rates of 2 × 10^10^ K/s and 4 × 10^10^ K/s. In summary, increasing the cooling rate helps to reduce stress in the gradient solidification region. However, it will lead to the solidification microstructure becoming homogeneous, which will result in a certain degree of stress stratification.

## 4. Discussion

### 4.1. Effect of Temperature Distribution on Nucleation

In solidified metallic materials, the degree of crystallization and homogeneous internal microstructure will affect the macroscopic mechanical properties and mechanical response. Therefore, after analyzing the differences between homogeneous solidification and gradient solidification in terms of solidification behaviour, phase transformation, and stress distribution, the degree of crystallinity of the microstructure after solidification should be further compared and analyzed. The RDF analysis of iron can further elucidate the differences in the microstructural characteristics under varying solidification conditions. The wider the width of the peaks and the larger the integrated intensity of the peaks of the curve in the RDF plot, the higher the degree of amorphization [19].

**Figure 8:**
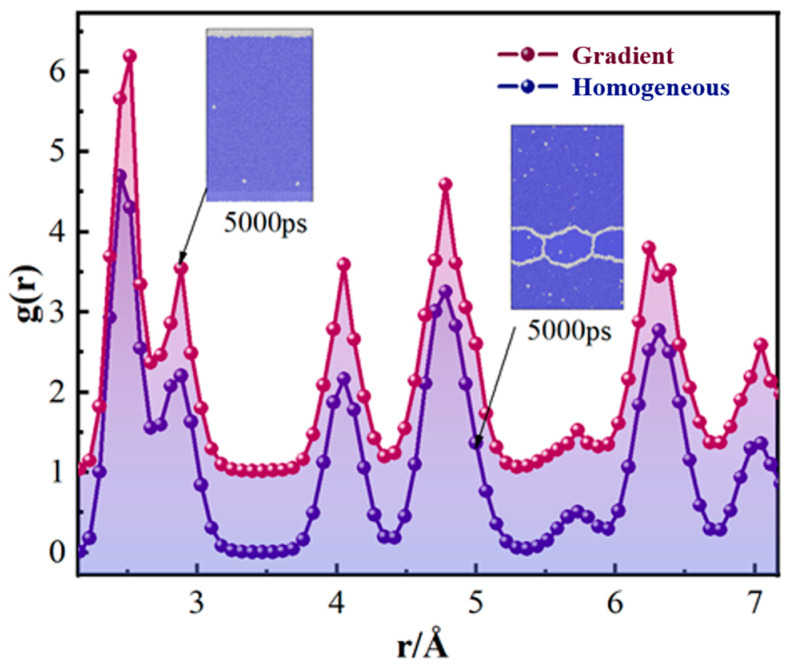
The RDF after homogeneous and gradient solidification.

Figure 8 shows the RDF curve after homogeneous and gradient solidification. It can be seen that under two different solidification conditions, the RDF curve will appear to have four independent and sharp peaks. However, after gradient solidification, the crest of the RDF curve is higher and sharper. This indicates that the degree of crystallinity is higher after gradient solidification [46]. Combined with the analysis of Figure 2d and Figure 3, it can be seen that the reason for this phenomenon is that the multiple crystal nucleus will be formed and grown at the same time during homogeneous solidification, resulting in the formation of many amorphous phases at the grain boundaries. In addition, the solidification efficiency of homogeneous solidification is high, resulting in part of the amorphous phase not completing the transformation. Therefore, compared with homogeneous solidification, iron forms a crystal structure with higher crystallinity after solidification at a specific gradient temperature.

The differences in latent heat release and nucleation rate between gradient solidification and uniform solidification can be further elucidated using classical nucleation theory (CNT). As shown in Equation (1), the critical nucleation energy ($\Delta G^{*}$) and thermal diffusion activation energy (Q) are important factors affecting the nucleation rate [47].

(1) $$I=I_{0}\exp\left(-\frac{\Delta G^{*}}{kT}\right)\exp\left(-\frac{Q}{kT}\right)$$

where I_0_ is a constant, T is the temperature, and k is the Boltzmann constant. The critical nucleation energy ($\Delta G^{*}$) can be obtained by Equation (2):

(2) $$\Delta G^{*}=\frac{16\pi \sigma_{\mathrm{SL}}^{3}T_{m}^{2}}{3{\left(\Delta H_{m}\Delta T\right)}^{2}}$$

where $\sigma_{SL}$ is the interface free energy, $\Delta H_{m}$ stands for the enthalpy of melting, T_m_ is the melting point, and $\Delta T=T_{m}-T$. As derived from Equation (2), the temperature difference within the gradient solidification model induces notable variations in the degree of undercooling (ΔT) and the interface free energy ($\sigma_{SL}$) compared to homogeneous solidification. Consequently, the critical nucleation energy ($\Delta G^{*}$) exhibits significant differences. Additionally, the thermal diffusion activation energy can be evaluated using the mean square displacement (MSD), whose slope varies with temperature [5]. It can be seen that these factors collectively contribute to pronounced differences in nucleation rates. In uniform solidification, the rapid nucleation rate causes the latent heat of crystallization to concentrate within a specific stage, resulting in a slight temperature increase, as illustrated in Figure 2d. Conversely, during gradient solidification, the slower nucleation rate facilitates the continuous release of latent heat, thereby avoiding abrupt temperature fluctuations within the system.

### 4.2. Effect of Cooling Rate and Temperature Gradient on Nucleation

**Figure 9:**
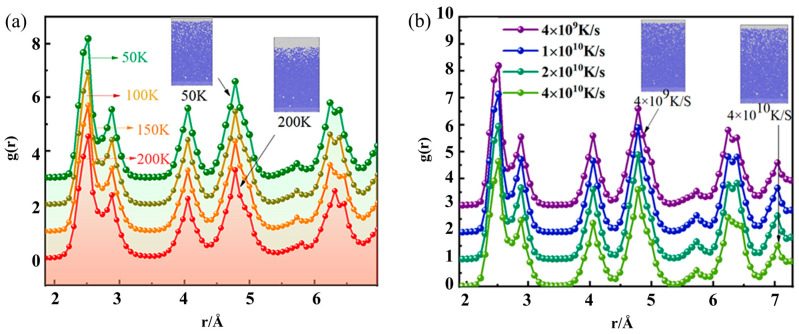
(**a**) RDF of solidified structures at different temperature gradients; (**b**) RDF of solidified structures at different cooling rates.

In order to further investigate the effect of different temperature gradients on the degree of crystallization during solidification, the RDF under different temperature differences was plotted, as shown in Figure 9a. During the gradient solidification, the waveforms all exhibit a series of sharp peaks at each temperature gradient. The four sharpest peaks are distributed near 2.51 Å, 4.05 Å, 4.78 Å, and 6.24 Å. This indicates a high degree of crystallization at the end of nucleation at all temperature gradients. It can be seen from the figure that the peak value of the first wave decreases as the temperature gradient increases. This indicates that the degree of grain size will decrease as the temperature difference increases. Therefore, the choice of temperature gradient should be combined with their actual needs and should not be too large.

The extent to which the cooling rate affects the crystallization of solidification is shown in Figure 9b. It can be seen that after solidification at each cooling rate, the RDF curves have four sharp and independent peaks at 2.5 Å, 4.05 Å, 4.78 Å, and 6.2 Å. In addition, the peak of the first wave decreases continuously as the cooling rate increases. This shows that the cooling rate gradually increases, and the overall degree of grain size will continue to decrease. Therefore, the cooling rate should not be too large.

## 5. Conclusions

In this study, the differences in solidification behaviour between homogeneous solidification and gradient solidification were systematically compared using MD theory with iron as the object of study. In addition, the solidification behaviour and the residual stress under different gradient temperatures and cooling rates are analyzed in depth. The main conclusions are as follows:

1. During homogeneous solidification, the crystals are transiently nucleated. When the temperature drops to 1431 K, the solid-state atoms aggregate into crystal nucleus and grow in all directions, finally forming a crystal microstructure with polycrystalline boundaries. During gradient solidification, nucleation occurs continuously, resulting in the gradual release of latent heat. In contrast, during homogeneous solidification, due to the shorter nucleation time, the crystallization latent heat is released over a short period. The BCC phase percentage inside the system increases slowly. The solid–liquid interface moves toward the region with a higher temperature. Compared to homogeneous solidification, the degree of crystallization increases by 24.07% after gradient solidification. However, compared to homogeneous solidification, the average and maximum stress values increased by about three and two times during gradient solidification.
2. The final BCC phase percentage after solidification is observed to be independent of the temperature difference. The increase in temperature difference leads to a more spatially disordered distribution of atoms, which in turn leads to a decrease in the crystallinity of the system. As the temperature difference is increased from 50 K to 200 K, the first peak RDF value of the solidification structure is observed to be reduced by about 12.30%. Furthermore, increasing the temperature gradient can effectively reduce the residual stresses in the solidified structure. The maximum and average stresses of the solidification structure are reduced by 30.29% and 38.04%, respectively.
3. The increase in cooling rate significantly enhances the conversion efficiency of the BCC phase. It is worth noting that higher cooling rates result in less crystallization of the microstructure after solidification. Increasing the cooling rate from 4 × 10^9^ K/s to 4 × 10^10^ K/s reduces the first peak RDF value by 11.85%. In addition, increasing the cooling rate results in a significant reduction in the stress level in the solidified microstructure.

## Data Availability

The original contributions presented in the study are included in the article, further inquiries can be directed to the corresponding author.

## Supplementary Materials

The following supporting information can be downloaded at: https://www.mdpi.com/article/10.3390/ma17246051/s1, Figure S1: Verification of the Simulation Model; Video S1: The structural transformation of iron during the homogeneous solidification. Reference [28] is cited in the Supplementary Materials.

## References

[1] Kurz W.F.D. (1989). Fundamentals of Solidification.

[2] Laaksonen A., Talanquer V., Oxtoby D.W. (1995). Nucleation: Measurements, Theory, and Atmospheric Applications. Annu. Rev. Phys. Chem..

[3] Reiss H., Kegel W.K., Katz J.L. (1998). Role of the Model Dependent Translational Volume Scale in the Classical Theory of Nucleation. J. Phys. Chem. A.

[4] Rein Ten Wolde P., Frenkel D. (1999). Homogeneous nucleation and the Ostwald step rule. Phys. Chem. Chem. Phys..

[5] Zhan L., Wu M., Qin X. (2021). Research on homogeneous nucleation and microstructure evolution of aluminium alloy melt. R. Soc. Open Sci..

[6] Tourret D., Liu H., Llorca J. (2022). Phase-field modeling of microstructure evolution: Recent applications, perspectives and challenges. Prog. Mater. Sci..

[7] Guillemot G., Gandin C.E., Combeau H.E. (2006). Modeling of Macrosegregation and Solidification Grain Structures with a Coupled Cellular Automaton—Finite Element Model. ISIJ Int..

[8] Iqbal N., Van Dijk N.H., Offerman S.E., Moret M.P., Katgerman L., Kearley G.J. (2005). Real-time observation of grain nucleation and growth during solidification of alumin-ium alloys. Acta Mater..

[9] Chen X., Fan W., Jiang W., Lin D., Wang Z., Hui X., Wang Y. (2022). Effects of Cooling Rate on the Solidification Process of Pure Metal Al: Molecular Dynamics Simulations Based on the MFPT Method. Metals.

[10] Pan K., Li Y., Zhao Q., Zhang S. (2019). Simulation of Solidification Process of Metallic Gallium and Its Application in Preparing 99.99999% Pure Gallium. JOM.

[11] Zhang T., Zhang X.R., Guan L., Qi Y.H., Xu C.Y. (2004). The simulation of metal Cu in the melting and solidification process. Acta Metall. Sin..

[12] Qi Z., Wang F., Wang Y., Wang Y. (2023). Effects of pressure on microstructure evolution of liquid Fe–S–Bi alloy during rapid solidification: A molecular dynamics study. J. Mol. Graph. Model..

[13] Yildiz A.K., Celik F.A. (2017). Atomic concentration effect on thermal properties during solidification of Pt-Rh alloy: A molecular dynamics simulation. J. Cryst. Growth.

[14] Agudelo-Giraldo J.D., Arias-Mateus D.F., Gomez-Hermida M.M., Reyes-Pineda H. (2023). Structural analysis of Ni nanoparticles in thermal cooling by molecular dynamics. Bull. Mater. Sci..

[15] Li K., Khanna R., Zhang J., Li G., Li H., Jiang C., Sun M., Wang Z., Bu Y., Bouhadja M. (2019). Determination of the accuracy reliability of molecular dynamics simulations in estimating the melting point of iron: Roles of interaction potentials initial system configurations. J. Mol. Liq..

[16] Aliotta F., Giaquinta P.V., Pochylski M., Ponterio R.C., Prestipino S., Saija F., Vasi C. (2013). Volume crossover in deeply supercooled water adiabatically freezing under isobaric conditions. J. Chem. Phys..

[17] Kavousi S., Gates A., Jin L., Zaeem M.A. (2022). A temperature-dependent atomistic-informed phase-field model to study dendritic growth. J. Cryst. Growth.

[18] Asta M., Beckermann C., Karma A., Kurz W., Napolitano R., Plapp M., Purdy G., Rappaz M., Trivedi R. (2009). Solidification microstructures and solid-state parallels: Recent developments, future directions. Acta Mater..

[19] Xie L., Wu G., Liaw P.K., Wang W., Li D., Peng Q., Zhang J., Zhang Y. (2024). Temperature gradient enhances the solidification process and properties of a CoCrFeNi high-entropy alloy: Atomic insights from molecular dynamics simulations. Comput. Mater. Sci..

[20] Gan X., Xiao S., Deng H., Sun X., Li X., Hu W. (2014). Atomistic simulations of the Fe(001)-Li solid-liquid interface. Fusion Eng. Des..

[21] Deb Nath S.K., Shibuta Y., Ohno M., Takaki T., Mohri T. (2017). A Molecular Dynamics Study of Partitionless Solidification and Melting of Al-Cu Alloys. ISIJ Int..

[22] Gan X., Deng H., Xiao S., Li X., Hu W. (2015). The alloying processes in solid-solid and liquid-solid Li-Pb interfaces with atomistic simulations. J. Alloys Compd..

[23] Liu Y., Han Q. (2021). Interaction between nucleant particles and a solid-liquid interface in Al-4.5Cu alloy. Acta Mater..

[24] Yan R., Sun W., Ma S., Davidchack R., Di Pasquale N., Zhai Q., Jing T., Dong H. (2018). Structural and mechanical properties of homogeneous solid-liquid interface of Al modelled with COMB3 potential. Comput. Mater. Sci..

[25] Frolov T., Mishin Y. (2010). Orientation dependence of the solid-liquid interface stress: Atomistic calculations for copper. Model. Simul. Mater. Sci. Eng..

[26] Liu J., Davidchack R.L., Dong H.B. (2013). Molecular dynamics calculation of solid-liquid interfacial free energy and its anisotropy during iron solidification. Comput. Mater. Sci..

[27] Mendelev M.I., Han S., Srolovitz D.J., Ackland G.J., Sun D.Y., Asta M. (2003). Development of new interatomic potentials appropriate for crystalline and liq-uid iron. Philos. Mag..

[28] Waseda Y. (1980). The Structure of Non-Crystalline Materials: Liquids and Amorphous Solids.

[29] Verlet L. (1967). Computer “Experiments” on Classical Fluids, I. Thermodynamical Properties of Len-nard-Jones Molecules. Phys. Rev..

[30] Hoover W.G. (1985). Canonical dynamics: Equilibrium phase-space distributions. Phys. Rev. A.

[31] Hoover W.G. (1986). Constant-pressure equations of motion. Phys. Rev. A.

[32] Plimpton S. (1995). Fast Parallel Algorithms for Short-Range Molecular Dynamics. J. Comput. Phys..

[33] Monasse B., Pradille C., Chastel Y. (2014). A molecular dynamics simulation study of semi-solid-state Fe: High temperature elasticity and void formation in liquid. Metall. Res. Technol..

[34] Lai L.S., Wu Y.Q., Shen T., Zhang N., Gao S. (2012). Molecular dynamics simulation of induced solidification process of pure liquid fe by Al_2_O_3_ nanoparticles. Acta Phys.-Chim. Sin..

[35] Tomoya Y., Yasushi S., Munekazu O. (2025). Molecular dynamics simulation of excess vacancy formation during rapid solidification of pure metals. Comput. Mater. Sci..

[36] Stukowski A. (2010). Visualization and analysis of atomistic simulation data with OVITO–the Open Visualization Tool. Model. Simul. Mater. Sci..

[37] Faken D., Jónsson H. (1994). Systematic analysis of local atomic structure combined with 3D computer graphics. Comput. Mater. Sci..

[38] Xie L., Brault P., Thomann A.L., Yang X., Zhang Y., Shang G. (2016). Molecular dynamics simulation of Al–Co–Cr–Cu–Fe–Ni high entropy alloy thin film growth. Intermetallics.

[39] Okita S., Verestek W., Sakane S., Takaki T., Ohno M., Shibuta Y. (2017). Molecular dynamics simulations investigating consecutive nucleation, solidification and grain growth in a twelve-million-atom Fe-system. J. Cryst. Growth.

[40] Xu J., Xiang M., Dang B., Jian Z. (2017). Relation of cooling rate, undercooling and structure for rapid solidification of iron melt. Comput. Mater. Sci..

[41] Zhang Q., Wang J., Tang S., Wang Y., Li J., Zhou W., Wang Z. (2019). Molecular dynamics investigation of the local structure in iron melts and its role in crystal nucleation during rapid solidification. Phys. Chem. Chem. Phys..

[42] Shibuta Y., Sakane S., Miyoshi E., Okita S., Takaki T., Ohno M. (2017). Heterogeneity in homogeneous nucleation from billion-atom molecular dynamics simulation of solidification of pure metal. Nat. Commun..

[43] Liu X.J., Xu Y., Hui X., Lu Z.P., Li F., Chen G.L., Lu J., Liu C.T. (2010). Metallic Liquids and Glasses: Atomic Order and Global Packing. Phys. Rev. Lett..

[44] Heino P., Ristolainen E. (1999). Molecular dynamics study of thermally induced shear strain in nanoscale copper. IEEE Trans. Adv. Packag..

[45] Shibuta Y., Sakane S., Takaki T., Ohno M. (2016). Submicrometer-scale molecular dynamics simulation of nucleation and solidification from undercooled melt: Linkage between empirical interpretation and atomistic nature. Acta Mater..

[46] Shibuta Y., Oguchi K., Takaki T., Ohno M. (2015). Homogeneous nucleation and microstructure evolution in million-atom molecular dynamics simulation. Sci. Rep..

[47] Yu Y.N. (2000). Principles of Metal Science.

