# Supplementary material for: Effect of Temperature Gradient and Cooling Rate on the Solidification of Iron: A Molecular Dynamics Study
Source: Materials (Basel). 2024 Dec 11;17(24):6051. doi: 10.3390/ma17246051 (PMC11727800; doi:10.3390/ma17246051)
Supplement: Supplementary file 1 [file materials-17-06051-s001.zip › materials-2917427-supplementary.pdf]

materials

MDPI

Supplementary Information

Effect of Temperature Gradient and Cooling Rate on the Solidification of Iron: A Molecular Dynamics Study

Qin Qin, Weizhuang Li, Wenrui Wang, Dongyue Li * and Lu Xie *

School of Mechanical Engineering, University of Science and Technology Beijing, Beijing 100083, China
* Correspondence: lidongyue@ustb.edu.cn (D.L.); xielu@ustb.edu.cn (L.X.)

Figure S1: Verification of the Simulation Model

The selection of the EAM potential function and the modeling scheme in the simulation system significantly influences the reliability and accuracy of the solidification results. In this section, a validation model is constructed to assess its accuracy. First, an initial model containing 45,696 iron atoms is established. Subsequently, the system temperature is raised from 300 K to 2500 K over 50,000 steps. After relaxing and equilibrating the entire system for 500,000 steps, the solidified iron model is obtained. During the simulation, a time step of 0.001 ps is employed. When the model is cooled to 1650 K at a cooling rate of 100 K/s, the RDF curve is extracted and compared with the experimental values reported by Waseda[28].

As shown in Figure S1, the two curves exhibit excellent agreement, indicating that the simulation results reliably capture the microstructural evolution laws of the system.

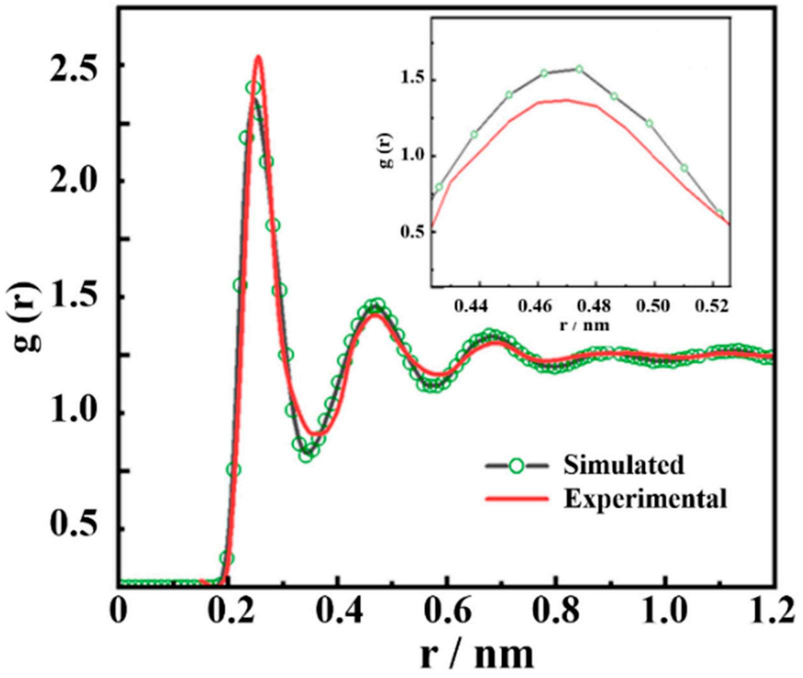

Figure S1. Comparison of the RDF values obtained by the simulation model and that in the reference [1].

Video S1: The Structural Transformation of Iron During the Homo-Geneous Solidification.

The structural transformation of iron during the homogeneous solidification (the blue atoms represent the BCC phase, and the gray atoms represent the amorphous phase).

Reference

1. Waseda, Y. The Structure of Non-Crystalline Materials: Liquids and Amorphous Solids; McGraw-Hill International Book Co.: New York, NY, USA, 1980.
